# Loss of Xist RNA from the inactive X during B cell development is restored in a dynamic YY1-dependent two-step process in activated B cells

**DOI:** 10.1371/journal.pgen.1007050

**Published:** 2017-10-09

**Authors:** Camille M. Syrett, Vishal Sindhava, Suchita Hodawadekar, Arpita Myles, Guanxiang Liang, Yue Zhang, Satabdi Nandi, Michael Cancro, Michael Atchison, Montserrat C. Anguera

**Affiliations:** 1 Department of Biomedical Sciences, School of Veterinary Medicine, University of Pennsylvania, Philadelphia PA, United States of America; 2 Department of Pathology, School of Medicine, University of Pennsylvania, Philadelphia, PA, United States of America; Florida State University, UNITED STATES

## Abstract

X-chromosome inactivation (XCI) in female lymphocytes is uniquely regulated, as the inactive X (Xi) chromosome lacks localized Xist RNA and heterochromatin modifications. Epigenetic profiling reveals that Xist RNA is lost from the Xi at the pro-B cell stage and that additional heterochromatic modifications are gradually lost during B cell development. Activation of mature B cells restores Xist RNA and heterochromatin to the Xi in a dynamic two-step process that differs in timing and pattern, depending on the method of B cell stimulation. Finally, we find that DNA binding domain of YY1 is necessary for XCI in activated B cells, as *ex-vivo* YY1 deletion results in loss of Xi heterochromatin marks and up-regulation of X-linked genes. Ectopic expression of the YY1 zinc finger domain is sufficient to restore Xist RNA localization during B cell activation. Together, our results indicate that Xist RNA localization is critical for maintaining XCI in female lymphocytes, and that chromatin changes on the Xi during B cell development and the dynamic nature of YY1-dependent XCI maintenance in mature B cells predisposes X-linked immunity genes to reactivation.

## Author summary

Females are predisposed to develop various autoimmune disorders, and the genetic basis for this susceptibility is the X-chromosome. X-linked genes are dosage compensated between sexes by X-chromosome Inactivation (XCI) during embryogenesis and maintained into adulthood. Here we show that the chromatin of the inactive X loses epigenetic modifications during B cell lineage development. We found that female mature B cells, which are the pathogenic cells in autoimmunity, have a dynamic two-step mechanism of maintaining XCI during stimulation. The transcription factor YY1, which regulates DNA looping during V(D)J recombination in B cells, is necessary for relocalizing Xist RNA back to the inactive X in activated B cells. YY1 deletion *ex vivo* in mature B cells impairs heterochromatin mark enrichment on the inactive X, and results in increased X-linked gene expression. We demonstrate that the DNA binding domain of YY1 is sufficient for localizing Xist RNA to the inactive X during B cell stimulation. Our study indicates that Xist RNA localization is critical for maintaining XCI in female lymphocytes. We propose that chromatin changes on the Xi during B cell development and the dynamic nature of YY1-dependent XCI maintenance in mature B cells predisposes X-linked immunity genes to reactivation.

## Introduction

Maintaining the proper dosage of gene expression is essential for the survival of all mammals. To balance the unequal number of sex chromosomes between females (XX) and males (XY), female mammals transcriptionally silence a single X-chromosome through the process of X-chromosome inactivation (XCI), thereby equalizing the expression of X-linked genes between the sexes [1, 2]. XCI is initiated during early embryonic development by expression of the long noncoding RNA Xist, which is transcribed from a single X-chromosome that will become inactivated [3–7]. Xist RNA transcripts function in *cis* as molecular scaffolds to recruit protein complexes for deposition of heterochromatic modifications, including H3K27me3, H2a-Ubiquitinylation (H2a-Ub), the histone variant macro-H2a, and H4K20me, across the chromosome, resulting in transcriptional repression [8–10]. The memory of the transcriptionally silent Xi is maintained with each cell division throughout the entire lifetime of somatic cells, so that dosage compensation of X-linked genes is faithfully preserved. Continuous expression of Xist RNA also plays a role in maintaining transcriptional repression of the Xi during the maintenance phase of XCI. Human pluripotent stem cells that have irreversibly silenced *XIST* lack enrichment of heterochromatic marks on the Xi, and exhibit overexpression of X-linked genes [11–13]. Deletion of *Xist* in hematopoietic stem cells also compromises XCI maintenance in the blood lineages, and increased expression of X-linked genes in female mutant mice results in hyper-proliferation and development of myeloid cancers [14]. X-linked genes are frequently overexpressed in female lymphocytes from lupus patients and mouse models of autoimmunity [15–18], which suggests that XCI may be perturbed in these cells [19, 20].

The transcription factor Ying Yang 1 (YY1) contributes to the initiation and maintenance phases of XCI. YY1 is a multifunctional protein containing four zinc fingers that can either activate or repress transcription [21–23]. The *Xist* promoter region contains YY1 binding sites and YY1 enhances *Xist* expression during XCI initiation and maintenance in mammalian cells [24]. The YY1 protein can also bind RNA [25, 26], and the Repeat C region of Xist contains three YY1 binding sites required for tethering Xist RNA to the chromatin of the Xi in post-XCI somatic cells [27]. YY1 mediates the long-distance DNA interactions required for V(D)J recombination of the immunoglobulin loci and for class switch recombination, and B cell specific YY1 deletion arrests B cell development at the pro-B cell stage [28–32]. We recently found that YY1 deletion or disruption in activated female T and B cells disrupts Xist RNA localization to the Xi with minimal impact on *Xist* expression [33], suggesting that YY1 may regulate XCI maintenance through mediating Xist RNA localization in lymphocytes.

The X-chromosome contains the highest density of immunity-related genes [34], and these genes are subject to XCI to ensure appropriate expression levels in somatic cells [35–37]. Surprisingly, despite their expression of Xist RNA, mammalian naive T and B cells do not localize Xist RNA and heterochromatic marks to the Xi [33]. However, these modifications are present on the Xi in activated cells. Here, we determine when epigenetic modifications are first lost from the Xi in the B cell lineage, and evaluate the molecular mechanism by which YY1 mediates the return of Xist RNA and heterochromatic modifications to the Xi for XCI maintenance during B cell activation. Together, these data elucidate a novel, lymphocyte specific mechanism for XCI maintenance and provide a foundational framework for understanding the origins of increased X-linked gene expression in female lymphocytes from autoimmunity patients.

## Results

### Xist RNA and heterochromatin marks are gradually lost from the Xi during B cell development

**Fig 1:**
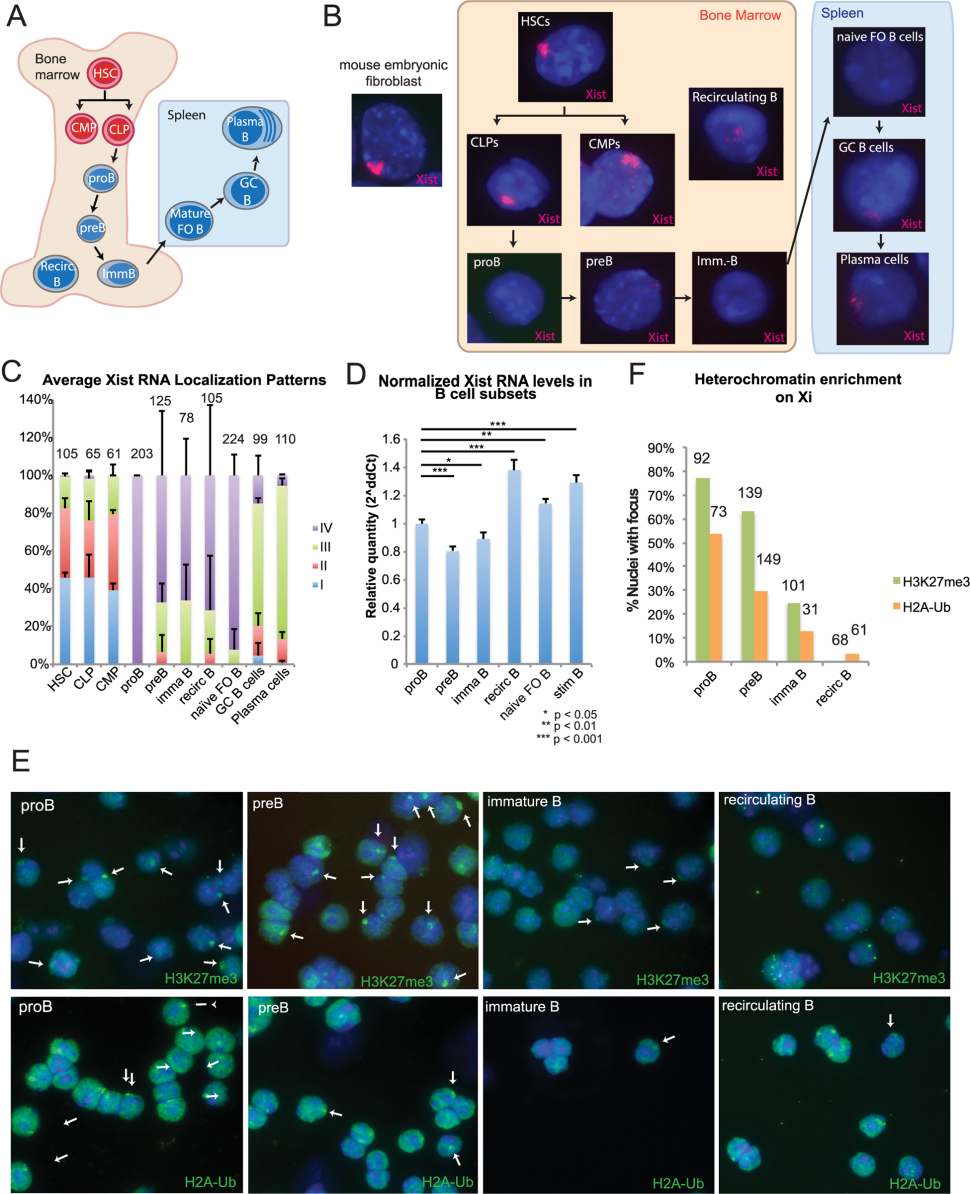
Xist RNA and heterochromatin marks are missing from the Xi during B cell development. (A) Schematic of B cell differentiation in bone marrow and spleen. Mature FO B cells, naïve FO B cells, and naïve B cells denote the same cell type, which are fully mature splenic B cells that have not seen antigen. (B) Representative Xist RNA FISH images of nuclei from each B cell subset. FACS plots are shown in Supplemental S1 Fig and described in Experimental Procedures. (C) Quantification of Xist RNA localization patterns from each B cell subset. The percentage for each pattern is averaged from two independent sorting experiments using non-immunized animals. Error bars denote standard deviations from the mean between replicate experiments. The total number of nuclei counted is shown above each column. (D) Relative quantity of Xist RNA in B cell progenitors from bone marrow and mature B cells from spleen; representative results from one experiment are shown here and results from replicate experiments are shown in S4 Fig. The housekeeping gene RPL13A was used for normalization. Statistical significance was determined using one-way ANOVA with post-hoc Tukey HSD test. Error bars denote standard deviations from the mean for one experiment. (E) Representative field images for immunofluorescence detection of H3K27me3 (top) and H2a-Ub (bottom) in developing B cells. White arrows denote heterochromatic foci for each modification. (F) Quantification of nuclei containing a heterochromatic focus from (E). The total number of nuclei counted is shown above each column.

We previously observed that mature splenic and recirculating B cells lacked canonical Xist RNA clouds and heterochromatic modifications [33]. Thus, we determined when Xist RNA first disappears from the Xi during B cell development (Fig 1A). To answer this question, we isolated hematopoietic stem cells (HSCs; Lin^-^, IL-7Ra^-^, c-kit^+^, Sca-1^+^), common lymphoid progenitors (CLPs; Lin^-^, IL-7Ra^+^, c-kit^+/lo^, Sca-1^lo^) and common myeloid progenitors (CMPs; Lin^-^, IL-7Ra^-^, c-kit^+^, Sca-1-, FcγR^lo^, CD34^+^) from bone marrow of female mice using fluorescence activated cell sorting (S1A Fig) [38, 39]. Sorted cells were immediately fixed for Xist RNA fluorescence in situ hybridization (FISH) using labeled short oligo probes. We classified Xist RNA localization patterns into four groups: Type I cells have robust Xist RNA localization on the Xi; Type II cells exhibit diffuse Xist RNA signals within a nuclear region encompassing the X-chromosome; Type III cells have Xist RNA pinpoints across the nucleus; Type IV cells lack Xist RNA signals (S2 Fig) [33]. We found that HSCs, CLPs, and CMPs exhibited Type I and II Xist RNA patterns, similar to fibroblasts, which are typically 80–90% Type I (Fig 1B and 1C and S3 Fig). Next, we examined CLP-derived B cell progenitors in the bone marrow, (Fig 1A), including pro-B cells (B220^lo^, AA4.1^+^, CD19^+^, CD43^+^), pre-B cells (B220^lo^, AA4.1^+^, CD19^+^, CD43^-^, CD23^-^, IgM^-^), and immature B cells (B220^lo^, AA4.1^+^, CD19^+^, CD43^-^, CD23^+/-^, IgM^+^) from female mice [40, 41] (S1B Fig), and then performed Xist RNA FISH. Remarkably, we found that pro-B cells completely lacked any Xist RNA signals (Fig 1B and 1C and S3 Fig). Pre-B cells had Type II and III diffuse patterns in about 30% of the cells, yet the majority of the cells lacked a detectible Xist RNA signal (Type IV) (Fig 1C). Immature B cells also mostly lacked Xist RNA signals, and had similar levels of Type III patterns (35%) as pre-B cells (Figs 1C and S3). We also examined recirculating mature B cells in bone marrow (B220^hi^, AA4.1^-^, CD19^+^, CD23^+^) (S1B Fig) and observed predominately faint and diffuse Type IV and Type III Xist RNA patterns (Fig 1C, and S3 Fig). Finally, we isolated germinal center (GC) B cells and plasma cells from spleens of non-immunized female mice (S1C Fig), and found that the Xist RNA localization patterns were predominantly Type III yet contained some Type I and II Xist RNA patterns (Fig 1B and 1C). In summary, Xist RNA disappears from the Xi at the pro-B cell stage and is absent through mature B cells, and is partially restored in a subset of cells (see below) because some GC and plasma cells are likely activated.

We hypothesized that the absence of robust Xist RNA ‘clouds’ on the Xi in developing B cell subsets could result from reductions in *Xist* expression. To address this, we examined the steady-state levels of Xist RNA in HSCs, CLPs, pro-B, pre-B, immature B, and recirculating mature B cells from bone marrow, and in naïve and *in vitro* stimulated mature splenic B cells (S4A Fig). We detected *Xist* expression in all developing B cell subsets, including lymphocyte progenitors with robust Xist RNA clouds, although the relative Xist RNA levels decreased slightly from the pro-B to the pre-B stage (*p* < 0.001; Fig 1D and S4B and S4C Fig). We also observed that Xist RNA transcripts were greater in recirculating B cells relative to immature B cells and earlier subsets (*p* < 0.001; Fig 1D and S4C Fig).

We next asked if the reduced *Xist* expression and lack of Xist RNA signals was accompanied by a reduction in heterochromatin marks on the Xi during B cell development. We performed sequential Xist RNA FISH followed by immunofluorescence to detect H3K27me3 and the ubiquitinylated histone H2A (H2A-Ub) in B cell subsets from bone marrow. We found that the majority of pro-B cells contained foci for both H3K27me3 and H2A-Ub (Fig 1E and 1F), despite the lack of Xist RNA transcripts localized on the Xi in these cells. Pre-B cells also exhibited foci for H3K27me3 and H2A-Ub which co-localized with an X-chromosome (S5A Fig), albeit at decreased levels compared to pro-B cells. Immature B cells had fewer nuclei with H3K27me3 (~25%) and H2A-Ub (~15%) foci (Fig 1E and 1F). Recirculating B cells from bone marrow had the fewest nuclei with foci for these heterochromatin marks. We conclude that the Xi is dramatically restructured during female B cell development, and that loss of Xist RNA localization, together with reduced *Xist* expression, from the Xi initiates the gradual disappearance of heterochromatin modifications.

### Xist RNA relocalizes to the Xi in two phases in activated splenic B cells

**Fig 2:**
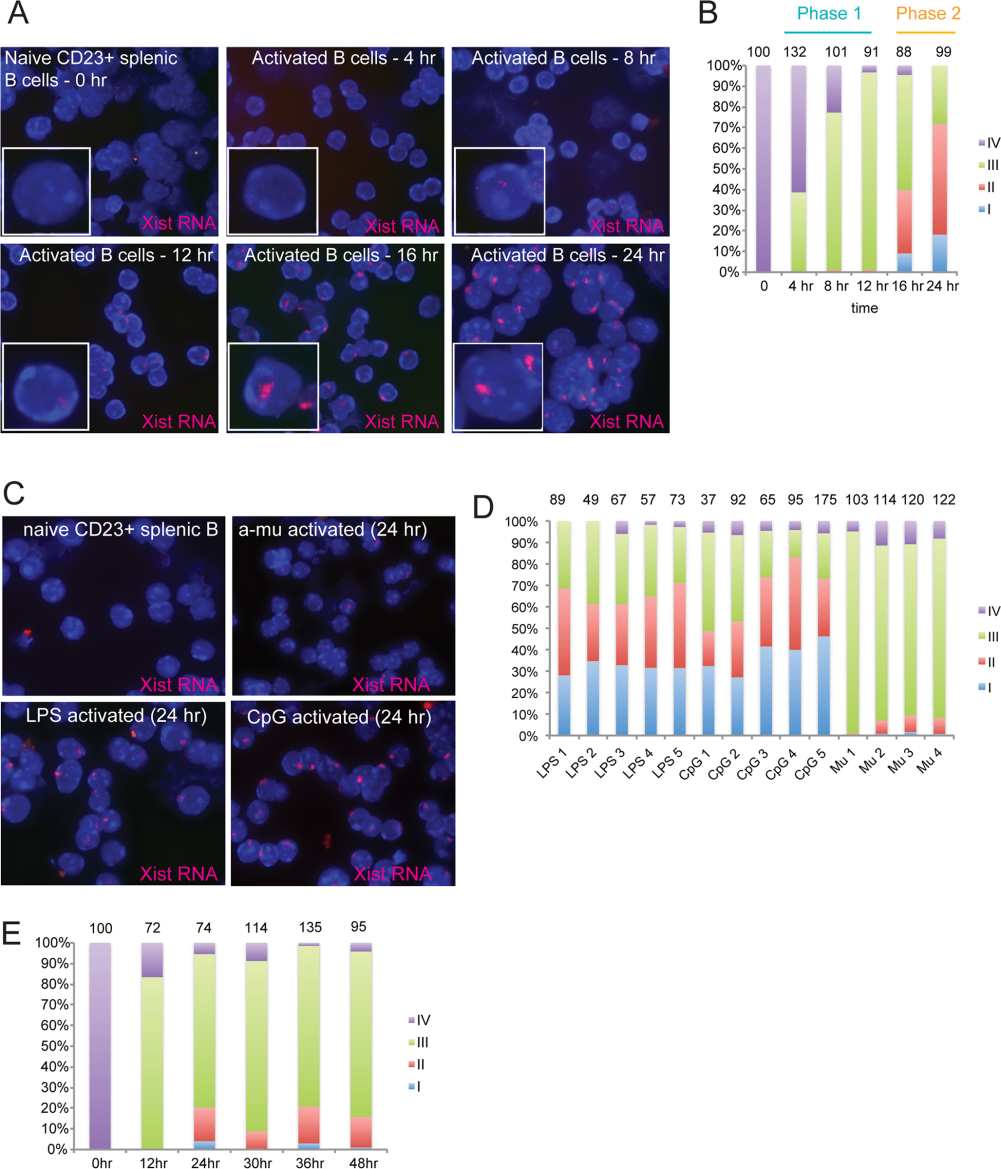
Timing of Xist RNA localization to the Xi during B cell stimulation. (A) Time course analysis, using RNA FISH, to monitor Xist RNA (red) localization changes immediately following B cell stimulation using CpG. Representative field images from one experiment are shown; similar results were obtained across 4 independent replicate experiments. (B) Representative results from one experiment quantifying the Xist RNA localization patterns for each time point after B cell stimulation using CpG. The total number of nuclei counted is shown above each column. Results from independent replicate experiments are shown in S6A Fig, along with statistical significance testing across all three experiments. (C) Representative Xist RNA FISH images of naïve splenic B cells and *in vitro* stimulated B cells (for 24 hrs) using three different stimuli. (D) Quantification of Xist RNA localization patterns for splenic B cells stimulated for 24 hrs using LPS, CpG, or anti-mu (Mu). Each column shows the average localization patterns for splenocytes from one female mouse. The total number of nuclei counted is shown above each column. (E) Time course analysis of splenic B cells stimulated with anti-mu. Representative results from one experiment quantifying Xist RNA localization patterns. The total number of nuclei counted is shown above each column.

Naïve female lymphocytes are the first physiological example of somatic cells where *Xist* is transcribed, yet Xist RNA does not localize to the Xi [33]. However, we have also shown that Xist RNA localization is restored in activated lymphocytes. To characterize the dynamic behavior of Xist RNA in activated lymphocytes, we performed a time course experiment to follow the visual appearance of Xist RNA transcripts on the Xi after *in vitro* stimulation of mature CD23^+^ follicular splenic B cells using CpG treatment. We performed RNA FISH in activated cells collected every 4 hours (Fig 2A). Unstimulated cells (0 hr) lacked detectible Xist RNA signals, consistent with a Type IV pattern. We observed Type III Xist RNA patterns, with pinpoints dispersed across the nucleus, starting at 4 hrs, and continuing to 12 hrs post stimulation (Fig 2A and 2B and S6A Fig). At 16 hrs post stimulation, Type I and II Xist RNA patterns appeared (Fig 2A and 2B and S6A Fig). Cells stimulated for 24–30 hrs have the highest percentages of Type I and II Xist RNA localization patterns (Fig 2A and 2B and S6B Fig). After 36 hrs, the percentage of Type I and II Xist RNA patterns were reduced 10–20% (S6B Fig). Xist RNA levels remained constant during the first 24 hrs of B cell stimulation (S7A Fig). In our experience, we have not observed differences with the kinetics of Xist RNA localization in activated B cells across different mouse strains (*C57Bl/6*, *BALB/c*, *129/SvJae*, *Mus Castaneus*) or in female mice from different ages (2–6 months). We conclude that Xist RNA localization to the Xi occurs in two distinct phases during B cell activation. During the first phase (Phase 1), Xist RNA transcripts cluster together across the nucleus (4–12 hrs post-stimulation), becoming visible as dispersed pinpoints with RNA FISH. In the second phase (Phase 2), occurring at 16–30 hrs post-stimulation, Xist RNA pinpoints localize to the Xi and form the bright and dense canonical Xist RNA ‘clouds’ in a localized nuclear territory at the periphery.

### B cell activation through the B cell receptor results in less efficient Xist RNA localization

In order to generate an immune response, B cells can be stimulated in different ways, utilizing distinct signaling pathways. Each of these methods exhibits differences with cell division rates, the timing of cell cycle progression, and propensity for differentiation into plasma cells, which influences cellular responsiveness of B cell subsets [42–44]. We investigated whether the activation method would affect the timing or distribution of Xist RNA localization patterns in mature splenic B cells. We stimulated splenic CD23^+^ B cells three different ways: (1) CpG DNA, a TLR9 agonist that induces cell proliferation [45, 46]; (2) lipopolysaccharide (LPS), which stimulates B cells through TLR4 signaling; or (3) anti-mu antibody, which signals through the B cell receptor (BCR) [47, 48]. We isolated splenic B cells from 5 different female mice (4 mice for anti-mu experiments), stimulated cells for 24 hrs, then quantified the localization patterns of Xist RNA using RNA FISH. We found that LPS and CpG stimulation had very similar distributions of Types I, II, III patterns (Fig 2C and 2D), yet CpG stimulation seemed to yield slightly more (3–5%) Type IV cells compared to LPS treatment (Fig 2D). Surprisingly, B cell stimulation using anti-mu resulted in predominantly Type III Xist RNA patterns (75–95%), with less than 10% of nuclei containing Type I or II clustered Xist RNA transcripts (Fig 2C and 2D). To determine if anti-mu induced alteration in Xist RNA localization was delayed relative to CpG and LPS, we assessed Xist RNA localization for 48–72 hrs after stimulation. We observed that Type III patterns predominate across all timepoints, with very few Type I cells and 10–20% Type II cells (Fig 2E). By 72 hrs post-stimulation, the percentages of Type I/II Xist RNA patterns were reduced for all three methods of B cell activation (S6C Fig). We conclude that B cell activation using CpG or LPS generated the highest amount of Type I/II Xist RNA patterns, and that anti-mu stimulation results in predominantly poorly localized Type III Xist RNA nuclear distribution.

### Xist RNA transcripts are short-lived in splenic B cells

The stability of Xist RNA transcripts might influence its localization to the Xi in activated B cells, where long-lived Xist RNA would be predicted to remain associated with the Xi.

**Fig 3:**
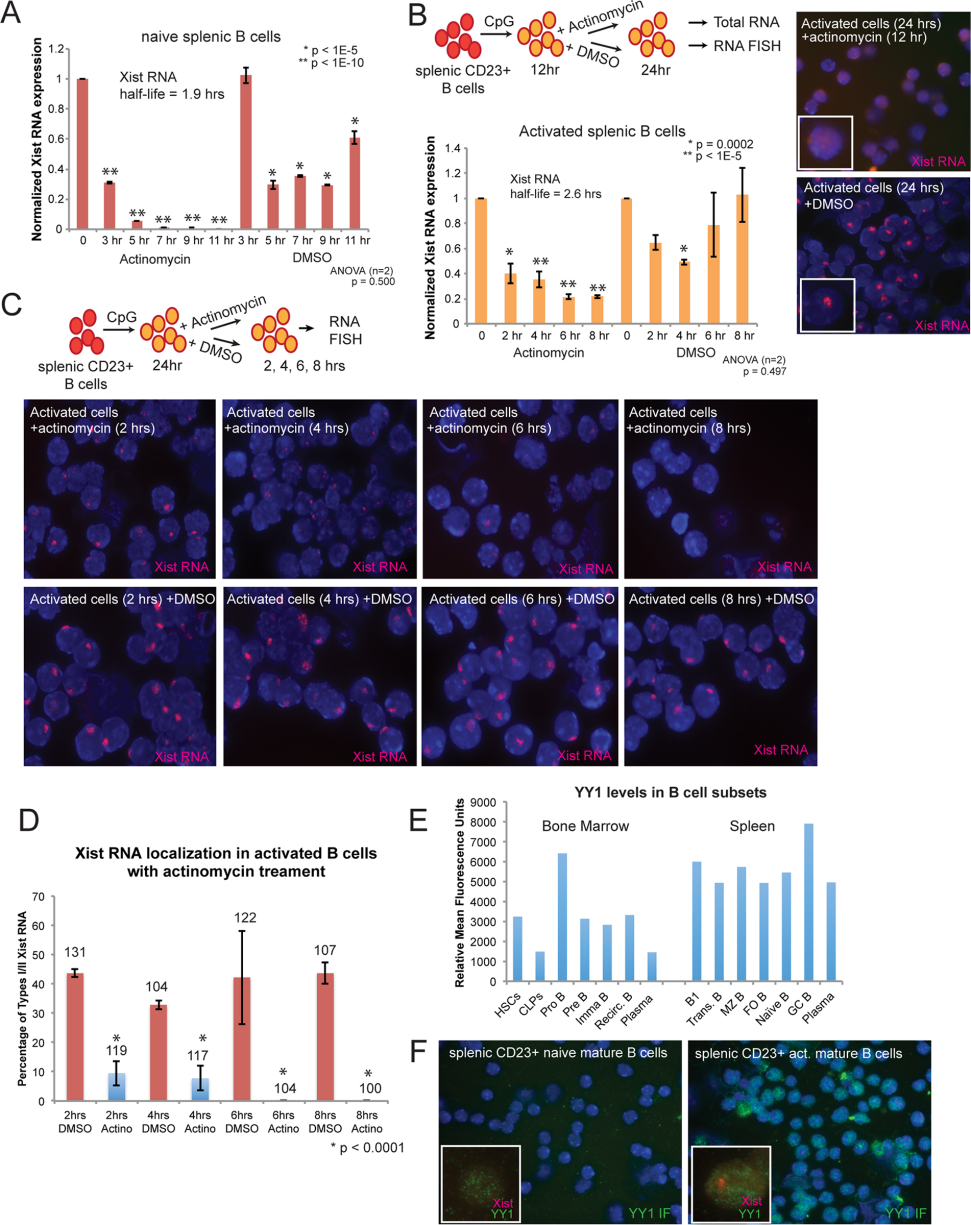
*Xist* is continuously transcribed in naïve and activated B cells, and YY1 is ubiquitous in all B cell subsets. (A) Xist RNA half-life measurement of naïve splenic B cells using actinomycin D or DMSO control. Xist RNA levels were normalized to the amount of transcript detected at time 0. Half-life is the amount of time for 50% of detectable Xist transcripts at time 0 to remain. Experiments were performed in duplicate, and cycling reactions in triplicate. Error bars denote standard deviations from the mean for technical replicates within one experiment. Statistical significance to test for reproducibility between independent replicate experiments was determined using ANOVA, and *p* values confirmed similar results. (B) Schematic for the experimental design to calculate Xist RNA half-life in activated splenocytes. Splenic B cells were stimulated with CpG for 12 hours, then treated with actinomycin D or DMSO control for another 12 hrs. Xist RNA half-life measurement of stimulated splenic B cells using actinomycin D, or DMSO control. Representative Xist RNA FISH images of activated splenic B cells after 12 hrs actinomycin treatment (or DMSO control). Error bars denote standard deviations from the mean for technical replicates within one experiment. Statistical significance to test for reproducibility between independent replicate experiments was determined using ANOVA, and *p* values confirmed similar results. (C) Schematic for time course experiments to examine Xist RNA localization following actinomycin treatment in activated B cells. Splenic B cells were stimulated with CpG for 24 hrs, then treated with actinomycin D or DMSO control for another 8 hrs, and cells were harvested every 2 hrs for Xist RNA FISH. Representative Xist RNA FISH images for activated B cells after 2, 4, 6, or 8 hrs of actinomycin treatment (or DMSO control). (D) Quantification of Type I/II Xist RNA localization patterns for actinomycin D and DMSO (control) treated cells. The average from two independent experiments is shown; error bars denote standard deviation from the mean between independent experiments. Statistical significance was determined using Chi-squared tests. The total number of nuclei counted is shown above each column. (E) Relative mean fluorescence intensity for YY1 protein in sorted B cell subsets (See Supplemental Experimental Procedures for cell surface markers). (F) Sequential Xist RNA FISH (red) and YY1 immunofluorescence (green) in naïve and CpG stimulated splenic B cells.

First, we investigated the half-life (t_1/2_) of Xist RNA transcripts in mature naïve B cells. We isolated splenic CD23^+^ B cells from female mice, treated cells with actinomycin D to inhibit RNA transcription for 0–11 hr, isolated RNA every 2–3 hr within this time period, and then used quantitative RT-PCR (qRT-PCR) to quantify Xist RNA (the housekeeping gene RPL13A was used for normalization). We found that Xist RNA transcripts rapidly decreased with actinomycin D treatment compared to DMSO treatment alone (Fig 3A), with a t_1/2_ of 1.9 hrs in mature naïve splenic B cells. This is significantly shorter than the t_1/2_ for Xist in female embryonic stem cells (ESCs) (~ 6 hrs) and female mouse embryonic fibroblasts (MEFs) (~ 3–4 hrs) [49]. To calculate the t_1/2_ for Xist RNA in CpG-stimulated splenic B cells, we treated activated B cells at 12 hrs post-stimulation with actinomycin for 8 hrs, then collected RNA at 2 hr intervals for qRT-PCR (Fig 3B). We found that Xist RNA has a t_1/2_ of 2.6 hrs in activated B cells, similar to naïve B cells. Because Xist RNA has a short half-life in mature B cells, we asked whether any Xist RNA that had become localized to the Xi at either 12 or 24 hrs post-stimulation would have been preferentially stabilized at the Xi. To address this question, we performed Xist RNA FISH using activated B cells treated with actinomycin or DMSO (control) for 2–12 hr intervals (Fig 3B and 3C). Significant levels of Xist RNA mislocalization from the Xi occurred after 2 hrs of actinomycin treatment, and no Type I/II patterns were present after 6 hrs (Fig 3D), indicating Xist RNA localization does not preferentially stabilize transcripts. Thus, Xist RNA is short-lived in B cells, and its localization to the Xi requires continuous transcription.

### H3K27me3 enrichment on Xi coincides with the relocalization of Xist RNA transcripts

**Fig 4:**
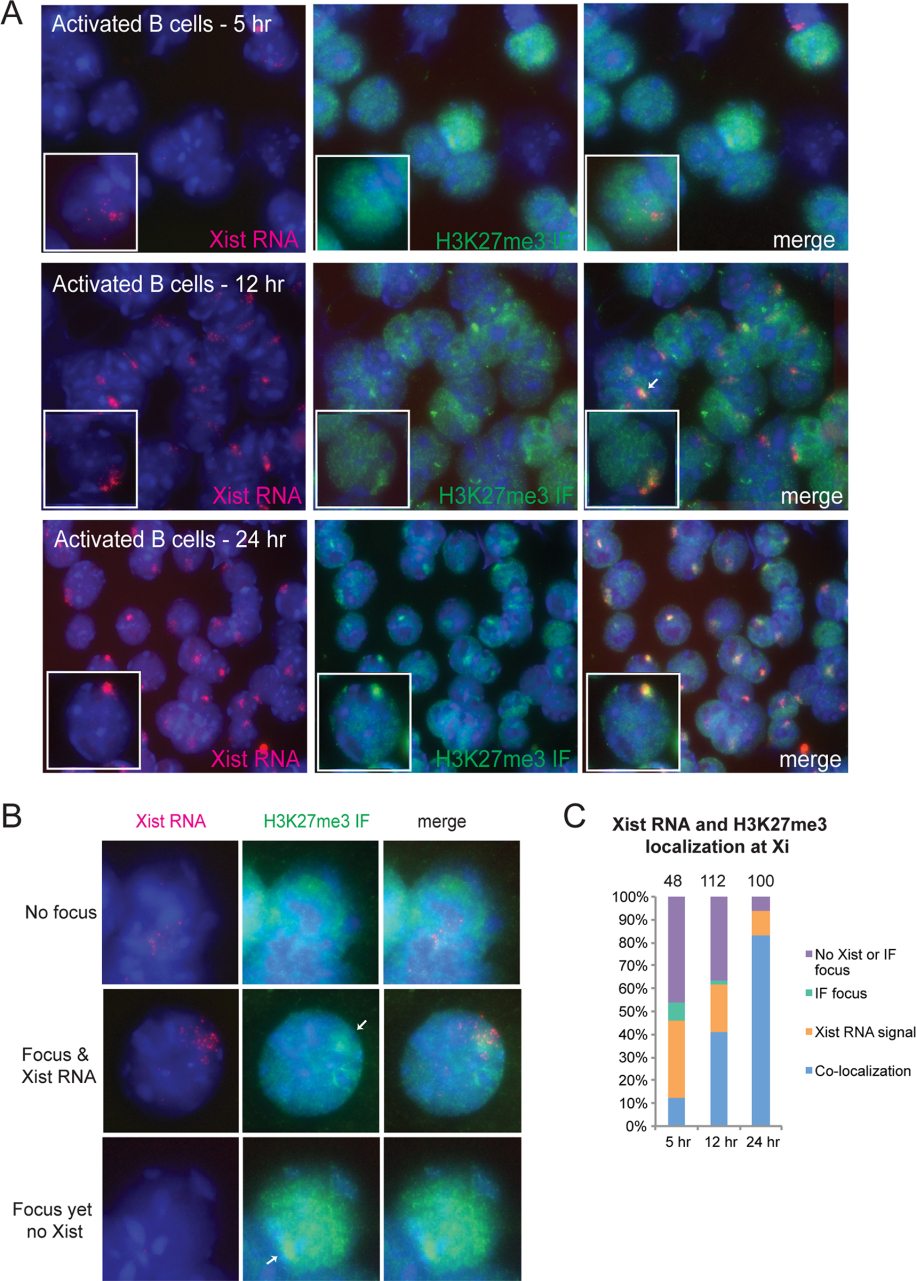
Simultaneous return of Xist RNA and H3K27me3 enrichment at the Xi during B cell activation. (A) Sequential Xist RNA FISH (red) then immunofluorescence detection (green) for H3K27me3 at 5, 12, 24 hrs post-stimulation. Representative results from one experiment (#1) are shown; results from experiments #2, 3 are shown in S8 Fig. One-way ANOVA analyses comparing all three experiments were not statistically different. (B) Examples of individual nuclei displaying different co-localization patterns of Xist RNA (red) and Xi heterochromatin (H3K27me3, green); taken 5 hrs post-stimulation. White arrows denote heterochromatic foci. (C) Quantification of co-localization patterns for Xist RNA and H3K27me3 (blue bars), Xist RNA signals alone (orange), H3K27me3 foci alone (green), or nuclei without either signals (purple) during B cell stimulation. Xist RNA signals corresponded to Type I, II, or III patterns. Results from one experiment are shown; three independent experiments were performed and these results are shown in S8 Fig together with statistical tests across all three independent replicates. The total number of nuclei counted is shown above each column.

As Xist RNA transcripts dynamically relocalize to the Xi during the first 24 hrs following B cell stimulation, we determined if such co-localization coincided with the appearance of H3K27me3 enrichment on the Xi. We activated splenic B cells with CpG, then performed sequential Xist RNA FISH followed by H3K27me3 IF for cells at 5 hrs, 12 hrs, and 24 hrs post-stimulation. We quantified the number of cells containing an Xist RNA signal (not distinguishing between Types I, II, or III), the number of cells containing an H3K27me3 focus, and cells with co-localization of both signals. Cells at 5 hours post-stimulation had strong H3K27me3 signals spread throughout the nucleus (Fig 4A), consistent with limited transcriptional activity of naïve B cells. At 5 hrs post stimulation, more than half of the cells lacked the colocalized Xist RNA/H3K27me3 signal (Fig 4A and 4C and S8 Fig; purple bars), typically observed in activated B cells and female fibroblasts. However, we found some examples of cells with a Type II Xist RNA pattern that contained a H3K27me3 focus, and also a few cells with a H3K27me3 focus without Xist RNA signal (Fig 4B), suggesting that H3K27me3 is unlikely to function as an imprint to direct the return of Xist RNA back to the Xi during splenic B cell activation. Sequential IF followed by DNA FISH for detection of the X-chromosomes confirmed that H3K27me3 foci overlapped with a signal for an X-chromosome (S5B Fig). The number of activated B cells with co-localization of Xist RNA signals and H3K27me3 foci increased at 12 hr and 24 hrs post-stimulation (Fig 4C and S8 Fig; blue bars). Nuclei with Type I Xist RNA patterns had the most robust H3K27me3 foci, most notable at 24 hrs post-stimulation (Fig 4A). The timing of H3K27me3 focal enrichment during B cell stimulation suggests that: (1) Xist RNA transcripts are localized on the Xi before or at the same time as H3K27me3 deposition; and (2) H3K27me3 marks are enriched on Xi during the second phase of Xist RNA localization.

### YY1 protein abundance does not dictate the extent of Xist RNA localization to the Xi during B cell development nor in mature B cells

We previously showed that YY1 is necessary for the formation of Type I Xist RNA clouds in activated lymphocytes [33], thus we speculated whether YY1 protein or RNA levels would correlate with the disappearance of Xist RNA localization on Xi during B cell development. To address this question, we isolated uncommitted progenitor cells (HSCs, CLPs), developing B cell subsets (pro-B, pre-B, immature B) and recirculating B cells from bone marrow, and mature B cell subsets from spleen using female mice. We measured YY1 protein levels by flow cytometry and YY1 RNA levels using qPCR (Fig 3E and S9A Fig). Uncommitted progenitor cells (HSCs, CLPs) have predominantly Type I Xist RNA localization patterns, yet CLPs have less YY1 protein compared to HSCs (Fig 3E). YY1 RNA levels were relatively consistent across all B cell subsets (S9A Fig). We found that pro-B cells, which lack detectible Xist RNA signals (predominantly Type IV), have the greatest amount of YY1 protein among bone marrow derived cells. Among splenic B cell subsets, YY1 protein levels were higher overall compared to bone marrow derived cells, and germinal center B cells had the greatest amount of YY1 across all samples. Thus, there was no correlation between YY1 RNA or protein levels and Xist RNA localization patterns in developing B cells, indicating that decreased YY1 protein levels do not account for the disappearance of Xist RNA from the Xi during B cell development.

Because Xist RNA and heterochromatin marks are localized to the Xi in activated lymphocytes, we asked whether the nuclear distribution of YY1 would change during B cell activation. We examined the nuclear distribution of YY1 protein using IF in naïve and activated splenic B cells, and observed that activated splenic B cells had more YY1 signal compared to naïve B cells (Fig 3F), as described recently [50]. Consistent with this, we found that YY1 RNA levels increase after 4 hrs of stimulation, and remain constant through 24 hrs post-stimulation (S7B Fig). However, we observed some overlap between YY1 and Xist RNA signals, yet we did not observe dramatic focal enrichment of YY1 protein that co-localized with Type I/II Xist RNA patterns in activated splenic cells (Fig 3F). Activation of splenic B cells through the BCR (using IgM/anti-mu) or Tlr9 (using CpG) did not affect YY1 protein levels, determined by measuring fluorescence intensity from immunofluorescence staining (S9B Fig). We conclude that there are no changes with YY1 nuclear localization in activated B cells.

### *Ex vivo* YY1 deletion eliminates heterochromatin enrichment on Xi and increases X-linked gene expression

**Fig 5:**
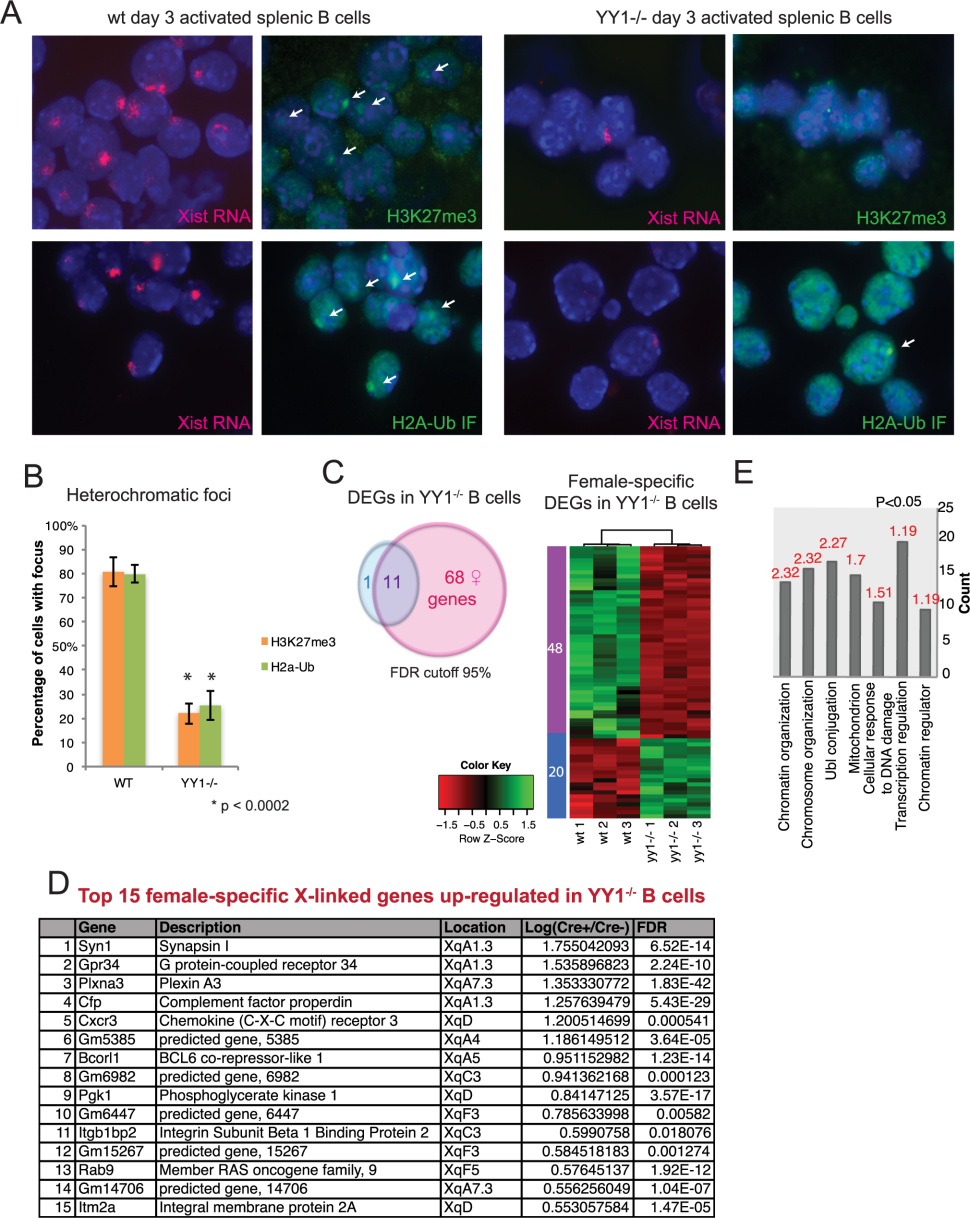
YY1 is required for Xi heterochromatin and X-linked gene dosage compensation in activated splenic B cells. (A) Sequential Xist RNA FISH (red) then immunofluorescence detection for H3K27me3 or H2a-Ub (green) in wild type (left) and *ex vivo* YY1 deleted (-/-; right) splenic B cells stimulated with CpG for 72 hrs. White arrows denote heterochromatic foci that overlap with an Xist RNA cloud. Representative results from one experiment are shown (n = 3). (B) Quantification of the number of nuclei in (A) containing a focus of heterochromatin. The averages from 3 experiments are shown, and statistical significance was determined using two-tailed t-test. Error bars denote standard deviations from the mean for biological replicates between independent experiments. (C) Sex-specific differentially expressed genes (DEGs) in YY1^-/-^ B cells. Venn diagram shows the comparison of RNA-seq results for unique male (blue) versus unique female (pink) YY1^-/-^ activated B cells. Shared DEGs shown in purple. Heat map shows hierarchical clustering of expression for the 68 female-specific X-linked transcripts for female wildtype and female YY1^-/-^ activated B cells. Cells were harvested two days after Cre-mediated deletion, corresponding to 48 hrs of stimulation. See S1 and S2 Tables for complete gene lists. Three independent mice for each condition (wildtype and YY1^-/-^) and sex were used for RNA isolation. (D) List of top 15 female-specific up-regulated X-linked genes in YY1^-/-^ B cells. Complete lists are shown in S1 Table. (E) Gene Ontology analysis for the complete list of X-linked genes upregulated in YY1^-/-^ B cells compared to female wildtype cells. Enrichment Score (ES) > 1, p < 0.05. The complete gene lists are shown in S2 Table.

We previously showed that disrupting expression of the transcription factor YY1 in T and B cells ablates Xist RNA localization to the Xi in activated cells [33]. We therefore next asked whether Xist RNA localization, acting through YY1-mediated recruitment, was necessary for the return of heterochromatin modifications to the Xi in activated B cells. We isolated CD23^+^ splenic B cells from wildtype and floxed YY1 female mice, treated the cells with recombinant TAT-CRE recombinase protein (to delete *Yy1*) [31], and stimulated the cells for 3 days using CpG. YY1 deletion after Tat-Cre treatment was confirmed at the RNA, DNA, and protein level (S10A Fig). Next, we performed sequential Xist RNA FISH followed by IF to examine heterochromatin enrichment on the Xi (Fig 5A). *Ex vivo* YY1 deletion greatly reduced the number of H3K27me3 and H2A-Ub foci in activated B cells from 80–85% to 20% (Fig 5B), and Type I and II Xist RNA patterns were also reduced (Fig 5A), as observed previously [33]. These results indicate that YY1-mediated Xist RNA localization to the Xi in activated B cells is strongly associated with the enrichment of H3K27me3 and H2A-Ub on this chromosome.

Next, we investigated whether the loss of heterochromatin marks on the Xi, resulting from YY1 deletion and the mislocalization of Xist RNA, would affect X-linked gene expression in female activated splenic B cells. We used *ex vivo* YY1 deletion in splenic XX or XY B cells from activated mice, with or without TAT-CRE, for RNA-seq analyses. Looking specifically at genes from the X-chromosome (manuscript in preparation for whole genome analyses of YY1 deletion), we determined the differentially expressed genes (DEGs) in Tat-Cre treated XX and XY samples (using FDR cutoff of 95%), removing the 11 genes present in both lists (Fig 5C). Next we compared the 68 female-specific DEGs (48 down-regulated; 20 up-regulated) between female wildtype and Tat-Cre treated B cells (Fig 5C; S1 Table, first tab). This list includes the immunity-related genes *Cxcr3* and *Itm2a* (Fig 5D), both of which are overexpressed in human female B cells relative to male B cells [33]. We also identified all of the DEGs comparing female wildtype to YY1-deleted cells, and found 211 X-linked upregulated genes, consistent with the loss of Xist RNA, H3K27me3, and H2A-Ub modifications from the Xi (S2 Table). Gene Ontology (GO) analysis of the 211 upregulated X-linked genes, comparing XX wildtype to YY1-deleted cells (S2 Table), revealed that affected pathways included chromatin and chromosome organization, mitochondrion, and also ubiquitin-like conjugation (Fig 5E). We also observed that 266 X-linked genes were down-regulated after *YY1* deletion in activated splenic B cells (S1 Table), including *Xist* (log2FC = -1.17) and neighboring genes *Jpx* (log2FC = -1.02) and *Ftx* (log2FC = -0.70), which activate *Xist* expression [51–53]. Such findings are expected, as YY1 is a transcriptional activator for *Xist* in post-XCI cells [24], thus *YY1* deletion would be expected to reduce Xist RNA levels and expression of target genes positively regulated by *Xist* expression. Using qPCR, we confirmed that Xist RNA levels decreased by 50% 48 hrs after Cre-mediated deletion (S10A Fig). In conclusion, YY1 is required to maintain XCI and transcriptional silencing of X-linked genes through regulating Xist RNA localization to the Xi.

### The YY1 DNA binding domain localizes Xist RNA transcripts to the Xi during B cell activation

**Fig 6:**
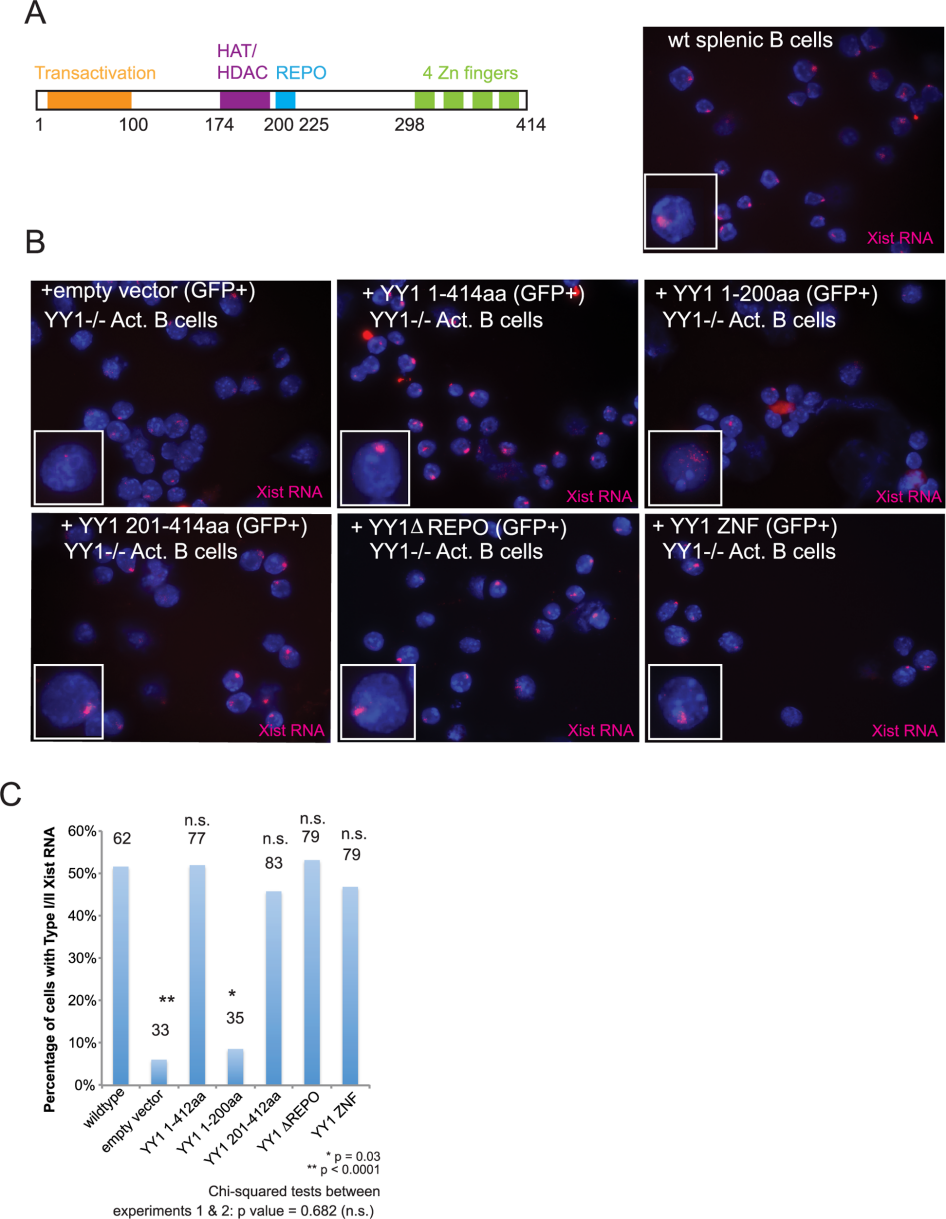
The C-terminal zinc finger domain of YY1 localizes Xist RNA to the Xi. (A) YY1 protein domains (left). A representative Xist RNA FISH image for wildtype LPS activated splenic B cells (right). (B) Rescue experiments in YY1^-/-^ B cells using various YY1 mutant proteins. YY1 was deleted using TAT-CRE protein, then cells were stimulated with LPS overnight and then infected with YY1 protein constructs co-expressing GFP. GFP positive and negative cells (S11 Fig) were sorted 48 hrs after transduction and then used for Xist RNA FISH. Clockwise from top left: empty vector; full length YY1 protein; N-terminal domain of YY1 (amino acids 1–200); C-terminal domain of YY1 (amino acids 201–414); full length YY1 lacking the REPO domain (deletion of amino acids 201–226); YY1 zinc finger domain (amino acids 290–414). Representative Xist RNA FISH (red) fields from one experiment are shown (n = 2). (C) Quantification of nuclei containing Type I and II Xist RNA localization patterns for a representative YY1 rescue experiment. The total number of nuclei counted is shown above each column. Statistical significance was determined using Chi-squared tests comparing wildtype to each rescue mutant for both experiments; p values are shown above total nuclei; n.s. not significant. One representative experiment is shown; similar *p* values for Chi-squared tests were obtained within each experiment and chi-squared tests between experiments 1 & 2 were not significantly different (p = 0.68) reflecting high reproducibility between experiments.

Because YY1 deletion impairs Xist RNA localization to the Xi in stimulated B cells, we next asked which protein domain(s) of YY1 were required for the localization (Fig 6A). To address this question, we deleted YY1 *ex vivo* in activated splenic B cells, then performed rescue experiments by adding back YY1 mutant proteins each missing distinct domains known to be critical for YY1 function. To select for transfected cells, human-versions of YY1 (hYY1) mutant proteins were co-expressed from a viral vector that also contained GFP with a separate internal ribosomal entry sequence (IRES). Expression levels (RNA and protein) for each hYY1 mutant after rescue were comparable (S10B Fig). We used FACS to sort infected cells (GFP+) from uninfected cells (GFP-) 48 hrs after infection for each hYY1 mutant, and performed Xist RNA FISH quantifying the percentage of nuclei with Types I or II Xist RNA patterns. Infection using an empty vector control resulted in predominantly Type III and IV Xist RNA patterns, with very few Type I/II cells (~3–5%; for 3 independent experiments) (Fig 6B and 6C), similar to our previous observations for YY1 deletion in splenic B cells [33]. Full-length hYY1 protein rescued Xist RNA localization to the Xi, with nearly identical levels of Type I/II Xist RNA patterns as wildtype B cells (Fig 6B and 6C). The N-terminal domain (NTD) of hYY1 (1–200 amino acids) contains the transcriptional activation domain and interacts with histone acetyltransferases and histone deacetylases [54, 55], as well as RNA binding activity that is independent of the zinc finger domain [26]. However, we found that expression of the 1–200 amino acid protein did not rescue the Xist RNA localization defect (Fig 6B and 6C). Next, we asked whether the C-terminal domain of hYY1 (CTD; 201–414 amino acids) that contains the REPO domain important for polycomb repressive complex 1 (PRC1) and polycomb repressive complex 2 (PRC2) interactions [56, 57] as well as four zinc fingers (ZNF) responsible for DNA binding activity [58] would rescue Xist RNA localization defects. HYY1 sequence 201–414 rescued Xist RNA localization to the Xi (Fig 6B and 6C), indicating that the critical domain for recruitment was located in this region. Surprisingly, we found that the DNA binding domain of YY1 alone, and not the REPO domain, was completely sufficient for Xist RNA localization in activated B cells, as expression of only the hYY1 four ZNF region was sufficient to rescue Xist RNA localization in activated B cells to wildtype levels (Fig 6B and 6C). As a control, we examined Xist RNA localization patterns for each sort of GFP-negative cells, and observed similar percentages of Type I/II Xist RNA patterns as empty-vector cells (S11 Fig). Importantly, expression of the hYY1 zinc finger construct in YY1 rescue experiments did not significantly alter *Xist* expression (S10C Fig), which supports a novel role for YY1 in Xist RNA localization separate from transcriptional activation. These results indicate that the YY1 DNA binding domain is critical for localizing Xist RNA to the Xi in activated splenic B cells.

## Discussion

**Fig 7:**
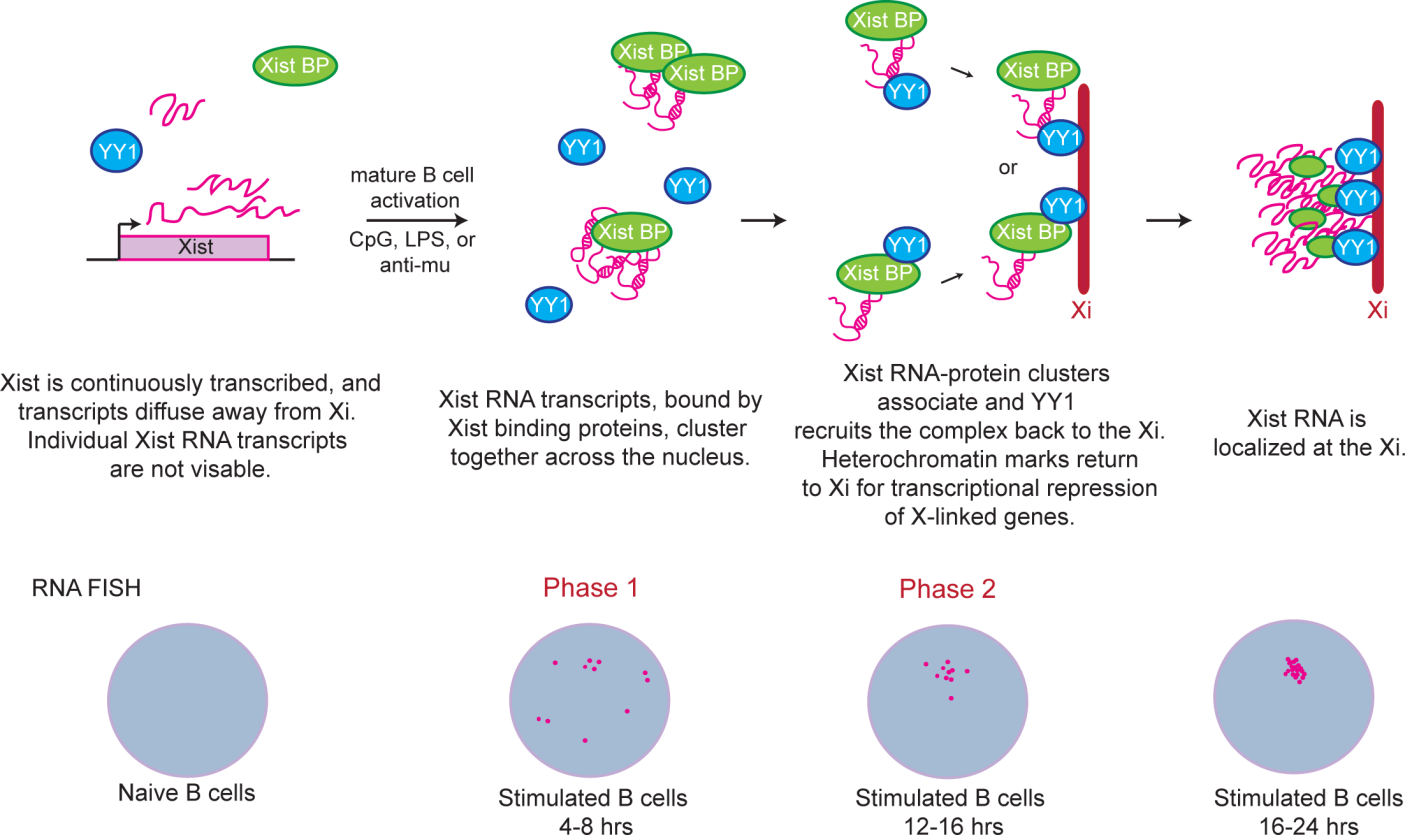
Model: Two phases for Xist RNA localization to the Xi during B cell stimulation. Naïve B cells lack detectible Xist RNA signals despite continuous *Xist* transcription. Xist RNA transcripts (pink) diffuse away from the Xi; YY1 protein is present, but at lower levels. During Phase 1 of Xist RNA localization (4–8 hrs post-stimulation), Xist RNA pinpoints are visible across the nucleus and RNA binding proteins cluster transcripts together to produce a visible signal by RNA FISH. During Phase 2 of localization (12–16 hrs post-stimulation), YY1 recruits Xist RNA transcripts, either directly binding RNA or indirectly interacting with Xist binding proteins (Xist BP), back to the Xi through the zinc-finger domain. Nuclei with robust Type I Xist RNA clouds have Xist RNA localized across the Xi.

Female mammals use XCI for dosage compensation of X-linked gene expression. However, in contrast to other somatic cells, lymphocytes use a unique and dynamic two-step mechanism to maintain XCI (Fig 7). In our study, we addressed the timing of Xist RNA loss from the Xi during B cell development. We found that Xist RNA is localized to the Xi in HSCs and CLPs, which display localization patterns similar to fibroblasts and other somatic cells. Intriguingly, Xist RNA is absent from the Xi beginning at the pro-B cell stage, and is missing from this chromosome throughout B cell development. Using sequential RNA FISH and IF, we demonstrated that the heterochromatin modifications H3K27me3 and H2A-Ub, usually enriched on the Xi, progressively disappear from the Xi during B cell development. Our observations of the presence of Xist RNA and H3K27me3 on the Xi for HSCs and CLPs, and the absence of these marks on pre-B, and immature B cells are in agreement with a previous study [59]. Our study indicates that the chromatin of the Xi progressively changes during B cell development, initiated with the loss of Xist RNA, and heterochromatin marks gradually disappear from the Xi as pro-B cells differentiate to immature B cells. Because Xist RNA is expressed in these cells, we propose that Xist RNA localization to the Xi, rather than *Xist* expression, is necessary to maintain enrichment of heterochromatin modifications on this chromosome, and that its absence on the Xi may initiate the chromatin reorganization of the Xi during differentiation of CLPs to pro-B cells.

Our work demonstrates that mature activated B cells, most notably *in vitro* stimulated follicular B cells, but to a lesser degree GC B cells and plasma cells, have robust Xist RNA clouds localized to the Xi. Some of the GC and plasma cells isolated from non-immunized mice were likely activated, which could be why we observe Type I and II patterns. Using TLR agonists to stimulate splenic B cells *in vitro*, we followed the nuclear locations of Xist RNA transcripts with RNA FISH over time. We found that Xist RNA relocalizes to the Xi in two steps, which we term Phase 1 and 2 (Fig 7). During Phase 1, occurring 4–12 hrs post-stimulation, Xist RNA transcripts are clustered and dispersed across the nucleus in the Type III pattern. We propose that Xist binding proteins, preferentially expressed in activated rather than naïve B cells, sequester Xist RNA transcripts, which are continuously expressed and likely diffuse away from the site of transcription (Fig 7). At present, the identity of Xist RNA binding proteins that could regulate Phase 1 of Xist localization during B cell stimulation is unknown. There are over 200 distinct RNA binding proteins that function in XCI initiation and maintenance in embryonic stem cells and MEFs [60–62], many of which function in chromatin remodeling and nuclear organization. We speculate that the Xist RNA pinpoints visible at 4–8 hrs post-stimulation are not individual Xist RNA molecules, which would have low signal intensity, but are instead clusters of Xist RNA nucleated by one or more RNA binding proteins, thereby generating a brighter fluorescence signal. Alternatively, Xist RNA tertiary structural changes may affect fluorescence detection during B cell stimulation.

In this study, we observed that Xist RNA signals become localized to nuclear regions encompassing the Xi during Phase 2 of localization, occurring 16–24hrs after stimulation, where the majority of cells exhibit Type I and II patterns. We found that YY1 is required for Phase 2 of Xist RNA localization, because YY1 deletion *ex vivo* results in accumulation of Type III cells and reduction of Type I Xist RNA localization patterns [33]. YY1 is a direct activator of *Xist*, and YY1 deletion *ex-vivo* reduced steady-state levels of Xist RNA about 2-fold (S1 Table; S10A Fig), which could contribute to the observed loss of heterochromatin mark enrichment at the Xi in stimulated B cells. It is possible that YY1 may also function for Phase 1 of Xist localization, because YY1 protein is still present during this time (4–12 hrs post-stimulation) in our *ex vivo* Tat-mediated deletion system. We propose that YY1 is one of several proteins that function in Xist RNA localization in stimulated B cells, as we recently described a similar function for hnRNP U in human T cells [33].

In Phase 2 of Xist RNA localization, Xist RNA can be recruited either directly or indirectly by YY1 to the Xi. YY1 can bind both DNA [21, 63] and also RNA, as Xist RNA can be immunoprecipitated using a YY1 antibody after UV crosslinking of female MEFs [27]. The RNA binding domain was recently mapped to the N-terminal domain excluding the zinc finger domain at the CTD [25]. However, another recent publication reported that the C-terminal zinc finger domain of YY1 can bind RNA [26]. In our study, we used a variety of different hYY1 mutant proteins to rescue Xist RNA localization in YY1 deleted splenocytes, and found that the zinc finger of YY1 is the only domain required for returning Xist RNA to the Xi (Fig 6). Importantly, ectopic expression of the hYY1 zinc finger domain (nor any of the hYY1 mutant proteins) did not affect Xist expression, indicating that YY1 transcriptional activation occurs independently of Xist RNA localization in activated B cells. Our results indicate that the DNA binding domain of YY1 may tether Xist RNA transcripts to the genomic DNA of the Xi. At present, it is unclear whether YY1 directly binds Xist RNA in activated splenocytes, or if YY1-interacting partners directly bind Xist RNA. YY1 interacts with various chromatin modifiers including Polycomb group proteins [56] and HDAC2 [64] in splenic B cells, and it is possible that YY1 tethers the Xist RNA-bound protein(s) to the Xi during Phase 2 of Xist localization in activated splenic B cells (Fig 7). The REPO domain of YY1 recruits Polycomb group proteins to DNA sequences [56], thus we were surprised that deletion of this domain still resulted in rescue of Xist RNA localization in YY1-deleted splenocytes. It is possible that that the EZH2 subunit of PRC2, which binds Xist RNA in fibroblasts and differentiating embryonic stem cells [10, 65], is directly recruited by Xist RNA to the Xi independently of YY1.

Our data demonstrate that H3K27me3 modifications co-localize with Type I and II Xist RNA signals as early as 5 hrs post-stimulation in activated splenic B cells. Because co-localization frequency increases with time post-stimulation, one interpretation is that Xist RNA becomes localized at the Xi at the same time as PRC2 is depositing H3K27me3 modifications across the Xi. It is well established that Xist RNA can bind to EZH2 subunit of PRC2 in MEFs and differentiating mESCs [10, 65], supporting a role for Xist RNA-mediated recruitment of PRC2 for localization at the Xi during B cell stimulation. However, at 5 hrs post-stimulation, we see evidence of some nuclei containing H3K27me3 foci that lacked Xist RNA (Fig 4B; bottom row). This observation suggests that there might be epigenetic modifications, perhaps DNA methylation, across the Xi that mark this chromosome for the return of H3K27me3 and H2A-Ub marks during B cell stimulation. DNA methylation patterns are an important marker that preserves the epigenetic state after cell division, where DNA replication produces a new daughter strand requiring modified nucleosomes to be recruited to maintain gene silencing [66–68]. It is possible that the DNA methylation modifications at CpG regions of promoters across the Xi do not change during B cell stimulation, and may serve as an imprint to direct the return of Xist RNA.

At present, it is unknown why the Xi loses heterochromatic modifications and Xist RNA during female B cell development, and how this might be beneficial for mature B cells. The X-chromosome is enriched for immunity-related genes, and there are some X-linked genes, such as *Btk* [69, 70], that are required for B cell development to continue beyond the pro-B cell stage. To our knowledge, X-linked genes are not required for V(D)J recombination, which occurs at the pro-B cell stage. We speculate that the euchromatic-like features of the Xi in developing B cells and the dynamic localization of Xist RNA and heterochromatin marks in activated mature cells might enable rapid reactivation of specific immune-related X-linked genes in response to pathogenic infections. Sex differences are observed in immune responses [71], and our study demonstrates that B cell development is different in females compared to male cells. Our findings provide a mechanism for the genetic basis for sex-biased autoimmune susceptibility involving B cells that overexpress immunity-related X-linked genes *TLR7* and *TLR8* [15–18, 72, 73].

In conclusion, our study provides a foundational framework that explains how X-linked genes can become over-expressed in female B cells. The chromatin of the Xi changes during the early stages of B cell commitment, beginning with the loss of Xist RNA localization at the Xi. Because YY1-mediated Xist RNA localization to the Xi during lymphocyte activation is a critical factor for regulating X-linked gene expression, we propose that monitoring changes in Xist RNA localization in female lymphocytes is an important parameter for diagnosing perturbations to XCI in female-biased autoimmune disorders involving pathogenic B cells.

## Materials and methods

### Ethics statement

Animal experiments were approved by the University of Pennsylvania Institutional Animal Care and Use Committee (IACUC). Euthanasia via carbon dioxide was used for animal sacrifice prior to spleen isolation.

### Murine B lymphocyte isolation and activation

Splenic B cells were isolated and from wildtype female mice (2–6 months) of various genetic backgrounds (C57BL/6, Balb/cJ, and 129S1), or from *yy1*^*flox/flox*^ mice on a C57BL/6 background (8–12 weeks). We did not observe differences with the kinetics of Xist RNA localization in activated B cells from different strains or ages. CD23^+^ mature follicular B cells were isolated using the MACS purification system with anti-CD23-biotin (eBioscience) and streptavidin microbeads (Miltenyi Biotec), and cultured as previously described (Wang et al., 2016). B cells were activated with 1 μM CpG (InvivoGen), 5 μg/mL LPS (Sigma), or 20 μg/mL anti-IgM.

### RNA FISH, DNA FISH, and immunofluorescence

Sequential RNA fluorescence in situ hybridization (FISH) and immunofluorescence (IF) was performed using established protocols [13, 74]. For Xist RNA FISH, two Cy3-labeled 20-nucleotide oligo probes were designed to recognize regions within exon 1 (synthesized by IDT) [33]. DNA FISH was performed with X-chromosome probes (MetaSystems Probes). For IF, cells we blocked with 0.2% PBS-Tween 0.5% BSA. Histone H3K27me2me3 (Active Motif; Cat. 39155) and Ubiquityl-histone H2a Lys119 (Cell Signaling; Cat. #8240) were diluted 1:100. Images were obtained using a Nikon Eclipse microscope and were categorized by types of Xist RNA localization patterns as described previously (Wang et al., 2016). Statistical significance was calculated using chi-squared tests and ANOVA.

### Xist RNA half-life analysis

Naïve and CpG activated splenic CD23^+^ B cells were treated with 5 μg/mL actinomycin D or DMSO. For activated cells, actinomycin D (Invitrogen) was added 12 or 24 hours after stimulation, and cells were harvested at various time points for both RNA FISH and qRT-PCR. RNA was isolated using TRIzol reagent (Invitrogen), and cDNA was synthesized with qScript cDNA SuperMix (Quanta). To determine the half-life, levels of Xist RNA were determined by qPCR using Power SYBR Green (Applied Biosystems) and the following Xist qPCR primer pair: 5’-CAGAGTAGCGAGGACTTGAAGAG-3’, and 5’-GCTGGTTCGTCTATCTTGTGGG-3’. Half-life experiments were performed twice, and the results from one representative experiment is shown. Samples were normalized to the level of Xist at time 0 for pre-actinomycin D treatment. Half-life was calculated using a linear regression equation, defining the amount of time (in hours) for half of the amount of Xist RNA (at time 0) to remain. Statistical significance was calculated using two-tailed t-tests and ANOVA.

### Isolation of developing B cell subsets for RNA FISH and qPCR analyses

Bone marrow or spleen cells were stained with antibodies for fluorescence activated cell sorting (FACS) analyses as previously described [50]. Briefly, cells were stained with fluorochrome-conjugated or biotinylated antibodies to mouse. Staining was done in PBS/1% BSA containing mouse IgG Fc fragments (Jackson Immunoresearch, Cat # 115-006-020). Dead cells and doublets were excluded and sorting was performed on a FACS Aria II machine using the following markers: Hematopoietic stem cells (HSC): Lin^-^(CD4[, CD8, B220, GR1, F4/80), IL-7R^-^, Sca-1^+^, c-kit^+^; Common lymphoid progenitors (CLP): Lin^-^, IL-7R^+^, Sca-1^lo^, c-kit^lo^; Common myeloid progenitors (CMP): Lin^-^, IL-7R^-^, Sca-1^-^, c-kit^+^, FcγR^lo^, CD34^+^; Progenitor B (Pro-B): B220^lo^, AA4.1^+^, CD19^+^, CD43^+^; Precursor B (Pre-B): B220^lo^ AA4.1^+^, CD19^+^, CD43^-^, CD23^-^, IgM^-^; Immature B (Imm-B): B220^lo^, AA4.1^+^, CD19^+^, CD43^-^, CD23^+/-^, IgM^+^; Recirculating mature B (Recirc-B): B220^hi^, AA4.1^-^, CD19^+^, CD23^+^; Follicular B (FO B): CD19^+^, B220^+^, AA4.1^lo/-^, CD43^-^, CD23^+^, CD21/35^lo/-^; B1-B: CD19^+^, B220^+^, CD43^+^; Transitional B: CD19^+^, B220^+^, AA4.1^+^, CD43^-^; Marginal zone B (MZ): CD19^+^, B220^+^, AA4.1^lo/-^, CD43^-^, CD23^lo/-^, CD21/35^+^; Plasma B: IgD^-^, DUMP(CD4, CD8, F4/80, GR1) ^-^, CD19^+/-^, CD138^hi^; Germinal Center B (GCB): IgD^-^, DUMP(CD4, CD8, F4/80, GR1) ^-^, CD19^+^, CD138^-^, GL7^hi^, FAS^hi^. Antibodies (Clone, Cat #) were purchased from BioLegend: CD19 (6D5, 115543), CD23 (B3B4, 101608), CD4 (RM4-5, 130312), CD8 (53–6.7, 100721), CD21/35 (7E0, 123418), CD43 (S11, 143204), IL-7R (A7R34, 135023), Sca-1 (D7, 108124), ckit (2B8, 105812), CD34 (MEC14.7, 119308), B220 (RA3-6B2, 103229), AA4.1 (AA4.1, 136510), IgD (11-26c.2a, 405725), F4/80 (BM8, 123112), FcγR (93, 101318), CD138 (281–2, 142516), eBiosciences: GR1 (RB6-8C5, 15-5931-82), and from BD Biosciences: IgM (R6-60.2, 562565), Fas/CD95 (Jo2, 557653). Data were analyzed using FlowJo software. We performed four independent sorting experiments using 2–5 C57BL/6mice for each sort (see S4 Fig), and the results and Ct values for all the experiments are shown in Figs 1 and S4. To determine Xist RNA expression levels in each B cell subset, we calculated the ratio of Ct differences relative to mature splenic naïve cells (Ratio = 2^ddCt), and used the housekeeping gene RPL13A [primer pair 5’-AGCCTACCAGAAAGTTTGCTTAC-3’ and 5’-GCTTCTTCTTCCGATAGTGCATC-3’] for normalization. We also used primers for another housekeeping gene (18S) for normalization, and obtained similar results as RPL13A. Statistical significance was determined using one-way ANOVA post-hoc with Tukey correction.

### Cre-mediated YY1 deletion *ex vivo* and rescue experiments with YY1 mutants

Splenic CD23^+^ B cells were purified from *yy1*^*flox/flox*^ animals (described above), then conditional YY1 knockout in splenic B cells was achieved using TAT-CRE enzyme purified from bacteria. Cells were washed three times with opti-MEM (Invitrogen) and then incubated with TAT-CRE for 45 min at 37°C. To inactivate TAT-CRE, fetal bovine serum was added to a final concentration of 10%. The cells were washed once with splenic B cell medium and then cultured at 37°C in a 5% CO2 atmosphere. The cells were activated *ex vivo* with 5 μg/ml LPS to stimulate proliferation [31]. Knockdown of endogenous YY1 was confirmed by qRT-PCR (primer pair: 5’-CGACGGTTGTAATAAGAAGTTTG-3’ and 5’-ATGTCCCTTAAGTGTGTAG-3’) and Western blots (sc414; Santa Cruz). After 24 hrs of YY1 deletion, cells were transduced with retrovirus supernatant containing either empty vector (pMX) or different human YY1-pMX constructs by 90 min infection. At 48 hours post infection, GFP positive and negative cells were isolated by FACS, and Xist RNA localization patterns were analyzed by RNA FISH. HYY1 levels were determined using human YY1-specific exonic primers within the CTD [primer pair: 5’-CACATGTGCGAATCCATACC-3’ and 5’-TGGTTGTTTTTGGCCTTAGC-3’] and also by Western blots, using an antibody for YY1 (sc414 Santa Cruz Biotechnology).

### YY1 mutant virus production and transduction

Human YY1 (hYY1), hYY1 1–200, hYY1ΔREPO, hYY1 zinc finger 288–414, and hYY1 201–414 sequences were cloned into the pMX-GFP vector BamHI-SnaB1 sites [32]. All hYY1 constructs were cloned in frame with GFP. High titer retrovirus supernatant was prepared using Fugene-6 (Promega) transfection into HEK293 cells along with retroviral envelope plasmid pHIT123. Viral supernatants were collected and concentrated with Retro-X-Concentrator (Clontech).

### Quantification of YY1 protein levels during B cell development

To determine levels of YY1, immunofluorescence was performed in naïve and CpG activated follicular B cells at an antibody dilution of 1:200 (Santa Cruz). Bone marrow and splenic cells were isolated from female mice (one mouse for each experiment), washed, and then stained for surface antigens in PBS with 2% bovine serum albumin (BSA) for 30 min at 4°C. Following washing, cells were treated with Cytofix/Cytoperm buffer (eBiosciences) and then stained for YY1 (clone EPR4652, Abcam) for 30 min at 4°C. Data were collected on a BD LSR II flow cytometer and analyzed with FlowJo software (Tree Star). The average fluorescence units were quantified for each cell subset and statistical significance was determined using two-tailed t-tests; representative results from one experiment is shown.

### RNAseq analyses of *ex vivo* deleted YY1^-/-^ from male and female splenic B cells

Mu-IgM/anti-CD40 stimulated follicular B cells were isolated from 3 month old female or male *yy1*^*flox/flox*^ animals (three separate animals each) treated with or without TAT-CRE. About 48 hours post-stimulation and YY1 deletion, total RNA was isolated using TRIzol (Invitrogen) and libraries were prepared with an Illumina TruSeq Stranded mRNA LT kit. Samples were run on Illumina NextSeq 500 and bioinformatic analyses were conducted as previously described [75]. Briefly, R (v3.3.1), RStudio (v1.0.44), and the Bioconductor suite of packages (http://www.bioconductor.org) were used to perform X-linked gene expression analyses. RNA-Seq reads were aligned with Tophat to the GRCm38/mm10 mouse reference genome and HTSeq-count and edgeR were used to normalize data. Statistical significance was determined with log2 fold change (lfc) and false discovery rates (FDR) (FDR <0.05, lfc > 0.5 or <-0.5). Heatmaps were constructed in RStudio (gplots, RColorBrewer), and expression levels for differentially expressed X-linked genes are shown. Gene Ontology (GO) term enrichment was performed using DAVID v6.8. A selection of Functional Annotation Clustering terms with an enrichment score >1 (p < 0.05) are shown. The RNAseq data are available in the Gene Expression Omnibus (GEO) database under the accession number GSE104097.

## Supporting information

S1 Fig Cell sorting of B cell subsets from bone marrow and spleen. (A) Representative FACS analysis for sorting hematopoietic stem cells (HSCs), common lymphoid progenitors (CLPs), and common myeloid progenitors (CMPs) from bone marrow. Female C57/Bl6 mice were used (n = 2; pooled); experiment was repeated twice.
(B) Representative FACS analysis for sorting pro-B, pre-B, immature B, and re-circulating B cells from bone marrow. Female C57/Bl6 mice were used (n = 2; pooled); experiment was repeated twice.
(C) Representative FACS analysis for sorting mature B cells, germinal center (GC) B cells, and plasma cells from spleens of female mice. Two female C57/Bl6 mice were used (pooled together); experiment was repeated twice.
(EPS)

S2 Fig Four types of Xist RNA localization patterns. Xist RNA FISH in CpG stimulated B cells (left column) and merged images with DAPI (center column). A cartoon representing each pattern is shown for each type.
(EPS)

S3 Fig Xist RNA FISH field images for B cell subsets from bone marrow. Xist RNA FISH for HSCs, CLPs, pro-Bs, pre-Bs, immature B cells, and recirculating B cells isolated from bone marrow. Type III Xist RNA patterns in pre-B and immature B cells are indicated with white arrows.
(EPS)

S4 Fig Real-time PCR experiments using B cell subsets and lymphocyte progenitors (HSCs, CLPs). (A) Schematic describing the four independent experiments for isolating B cell progenitors from bone marrow, RNA isolation, and technical parameters for qPCR. The housekeeping gene RPL13A was used for normalization. For experiments 1 and 2, bone marrow from two female mice were pooled for each experiment. For experiments 3 and 4, bone marrow from five female mice were pooled for each experiment.
(B) Ct values for Xist and RPL13A (housekeeping gene) for all four experiments.
(C) Relative quantity of Xist RNA in B cell subsets (shown is experiment 1; experiment 2 is shown in Fig 1) and lymphocyte progenitors (experiments 3 and 4). Samples of naïve and activated mature splenic B cells were included with each bone marrow isolation. Statistical significance was determined using one-way ANOVA with post-hoc Tukey HSD test, with pro-B cells and HSCs values set to 1. Error bars denote standard deviations from the mean for technical replicates within one experiment.
(EPS)

S5 Fig Co-localization of H3K27me3 foci with X-chromosomes for B cells lacking Xist RNA signals. Sequential IF (H3K27me3) then DNA FISH (X-paint) for the two X-chromosomes was performed. White arrows indicate H3K27me3 foci; white arrowheads denote locations for X-chromosomes.
(A) Two fields of pre-B cells; (B) mature splenic B cells 5 hrs post-stimulation.
(EPS)

S6 Fig Time course experiments for Xist RNA localization to the inactive X (Xi) during B cell activation. (A) Replicate experiments (#2, #3) for Xist RNA localization during the first 24 hrs of activation. One-way ANOVA analysis for each pattern of Xist RNA localization was tested across three independent experiments (exp. #1 shown in Fig 2), and p values were not significantly different, reflecting reproducibility of these results.
(B) Time course (0–48 hrs) of Xist RNA localization to the Xi after CpG stimulation of splenic B cells. Representative results from one experiment are shown (repeated twice). The total number of nuclei counted is shown above each column.
(C) Representative Xist RNA FISH images of naïve, LPS, CpG, and anti-mu/CD40 stimulated splenic B cells for 72 hours (left). Xist RNA localization patterns were quantified for each stimulation method (right).
(EPS)

S7 Fig Xist and YY1 RNA levels during B cell stimulation. Mature splenic B cells were isolated from two different female mice (mouse 1, mouse 2), then stimulated with CpG. Cells were collected every 4 hrs for RNA isolation, for qPCR analyses, and samples were normalized to the housekeeping gene RPL13A.
(A) Xist RNA levels during B cell activation. Two-tailed t-tests comparing mouse 1 and mouse 2 was not statistically significant (p = 0.324). Error bars denote standard deviations from the mean for technical replicates within one experiment.
(B) YY1 RNA levels during B cell activation. Two-tailed t-test comparing naïve B cells (0 hrs) to activated cells (24 hrs) were statistically different for both mice (p = 0.0008; p = 0.002). Error bars denote standard deviations from the mean for technical replicates within one experiment.
(EPS)

S8 Fig Co-localization of Xist RNA and H3K27me3 enrichment at the Xi during B cell activation. Sequential Xist RNA FISH (red) then immunofluorescence detection (green) for H3K27me3 at 5, 12, 24 hrs post-stimulation. Results from experiments #2, 3 are shown here (exp. #1 is shown in Fig 4), and one-way ANOVA analyses across all three experiments indicate reproducibility of the results.
(EPS)

S9 Fig Quantification of YY1 RNA and protein in B cell progenitors and during B cell activation. (A) qPCR analyses for YY1 RNA from lymphocyte progenitors and B cell subsets from bone marrow, and mature splenic B cells. Two-tailed t-tests were performed comparing HSCs (set to 1) for each independent experiment (performed twice). One-way ANOVA analysis comparing experiments 1 and 2 was not statistically significant, reflecting reproducibility with results. Error bars denote standard deviations from the mean for biological replicates between experiments.
(B) IF for YY1 protein in naïve splenic B cells and cells stimulated with IgM or CpG for 24 hrs. Fluorescence intensity for 10 nuclei from 6 different fields for each condition was quantified using Nikon Elements software. Statistical significance was determined using two-tailed t-tests (results are combination of two independent experiments); ns: not significant.
(EPS)

S10 Fig Tat-Cre mediated deletion (*ex vivo*) of YY1 in female splenic B cells. (A) (Top left) Quantification of YY1 protein via Western blots; B-actin was used a loading control. (Top right) Genomic DNA analysis of the mouse YY1 locus after Tat-Cre treatment for activated floxed YY1 splenic B cells using PCR amplification. The intact region produces a 369 bp band; deletion produces a 292 bp band. (Bottom) qPCR analyses for Xist RNA (left) and YY1 RNA (right) after Tat-Cre treatment. Two-tailed t-tests were used to compare mock vs tat-cre treated cells at both time points. One-way ANOVA analyses were used to test for reproducibility between two independent experiments (p = 0.563; p = 0.989). Error bars denote standard deviations from the mean for technical replicates within one experiment.
(B) (Top left) Rescue experiments using human YY1 mutants. Schematic for hYY1 mutants used in rescue experiments. (Top right) qPCR analysis for hYY1 mutant RNA expression in YY1-deleted splenic B cells. Exonic primers specific for hYY1 within the 201-414aa region were used; thus hYY1 1–200 RNA could not be detected. (Bottom left) Western blot analyses for hYY1 mutants transfected in 293T cells. Antibodies specific for YY1 or Flag-epitope were used to detect hYY1 mutants. Error bars denote standard deviations from the mean for technical replicates within one experiment.
(C) qPCR analysis for Xist RNA expression in hYY1 rescue experiments. Total RNA was isolated from activated YY1-deleted splenic B cells containing specific hYY1 mutants. Two-tailed t-tests were used to determine statistical significance, comparing empty PMX vector (set to 0.75) to each mutant construct. Error bars denote standard deviations from the mean for technical replicates within one experiment. The housekeeping gene RPL13A was used for normalization.
(EPS)

S11 Fig XIST RNA FISH of GFP-negative cells from YY1 rescue experiments. (A) Representative Xist RNA FISH images from GFP-negative cells for each YY1 mutant construct introduced into YY1^-/-^ activated splenic cells.
(B) Quantification of Type I/II Xist RNA localization patterns for each YY1 mutant used for rescue experiments in (A). Statistical significance was determined using Chi-squared tests, comparing wildtype to each hYY1 mutant. One representative experiment is shown; similar p values for Chi-squared tests were obtained within each experiment (repeated twice).
(EPS)

S1 Table Sex-specific differentially expressed genes in YY1^-/-^ B cells. Male-specific and female-specific gene lists are in separate tabs.
(XLSX)

S2 Table Complete list of differentially expressed X-linked genes comparing female wildtype to female YY1^-/-^ B cells. Each list (upregulated or down-regulated genes) is found in separate tabs.
(XLSX)
